# Airborne Ns (NO_2_ and NH_3_) in the Rijeka Bay Area (Croatia), 1980–1995

**DOI:** 10.1100/tsw.2001.276

**Published:** 2001-10-26

**Authors:** Ana Alebic-Juretic

**Affiliations:** Institute of Public Health, Rijeka, Croatia

**Keywords:** ambient levels, trend, emissions, nitrogen dioxide, ammonia

## Abstract

The determination of ambient levels of nitrogen dioxide (NO_2_) and ammonia (NH_3_) in the Rijeka Bay area started in 1980, as a part of the air quality monitoring programme. The results of 15 years of surveying (1980/81–1994/95) on ambient levels of these pollutants at two sampling sites are given in this work. Site 1 is located in the city, opposite the old petroleum refinery facilities, while Site 2 is located in the settlement 25 km from the city, opposite the eastern industrial zone. Annual means of NO_2_ varied between 34 and 60 μg/m3 at Site 1 and between 14 and 26 μg/m3 at Site 2, but do not follow the 40% reduction in industrial emissions of this pollutant, probably due to the dominant impact of other minor sources, like traffic. Yearly averages of NH3 were in the range of 13 to 26 μg/m3 at Site 1 and 7 to 16 μg/m3 at Site 2, and are practically constant during the period studied.

## INTRODUCTION

The products of $SO_2$, $NO_x$ (=NO + $NO_2$), and $NH_3$ emissions dominate the acidification of ecosystems. Dry and wet deposited $NH_3$ and $NH_4$ enter the soil layer as $NH_4^+$ where nitrification occurs resulting in acid formation[1]. Furthermore, both nitrogen oxides ($NO_x$) and $NH_3$ represent nitrogen inputs with eutrophy-cating effects on sensitive ecosystems. While industrial combustion and transport processes are the main sources of $SO_2$ and $NO_x$ emissions, livestock agricultural activities are dominant sources of $NH_3$ emissions in Europe. Other anthropogenic sources of $NH_3$ emissions include industrial $NH_3$ and fertilizer production, human sweat and breath, pets, sewage, landfill, coal combustion, and biomass burning[2]. Recent evidence suggests that people and traffic may have a greater impact in the nonagricultural regions, as ambient levels of $NH_3$ were found to be higher in more densely populated areas as compared to less densely populated areas[3].

The anthropogenic emission of $NO_x$ for 1990 was estimated at 31 million t N/a, with the contribution of fossil fuel combustion accounting for 70% of the total, and biomass burning for another 20%. The emission from natural sources (lightning and soil processes) was calculated at an additional 19 million t N/a. On the other hand, the global emission of $NH_3$ was estimated at 54 million t N/a, 70% of which is attributed to food production[4].

Rijeka is an industrialized city with approximately 200,000 inhabitants situated at the head of Rijeka Bay. The first industrial plants were founded in the 19th century at the edge of the city (e.g., the paper mill and the old petroleum refinery); the plants are nowadays located in the city centre, thus causing serious air pollution problems. During the 1960s new facilities of the petroleum refinery were erected in the eastern suburban industrial zone (Urinj), while some of the refining processes remained at the old location. During the 1970s a new oil-fired power plant and a coke plant were founded in the vicinity of the new refinery. Due to high emissions from these major industrial sources, the city of Rijeka came to be one of the most polluted cities in Croatia during the 1980s.

An air quality monitoring programme that started in mid-1970s included determination of ambient levels of $NO_2$ and $NH_3$ in the vicinity of industrial plants. The results of 15 years of surveying (1980/81–1994/95) of ambient $NO_2$ and $NH_3$ levels at two sampling sites within the Rijeka Bay area are presented in this work.

## MATERIALS AND METHODS

The location of sampling sites is given in Fig. 1. Site 1 is situated within the city area, above the busy street that divides the old petroleum refinery from the residential area; the site is affected by emissions from this industrial plant. Site 2 is located in the settlement 25 km southeast of Rijeka, opposite the new petroleum refinery facilities, oil-fired power plant, and coke plant. (The latter closed down in 1994.)

Average daily concentrations of $NO_2$ were determined by the modification of the Saltzman method[5], while the daily $NH_3$ concentrations were determined spectrophotometrically[6]. The sampling year comprised the period from October 1 through the following September 30.

## RESULTS AND DISCUSSION

$NO_x$ and $NH_3$ are involved in several chemical reactions in the troposphere. The gasses themselves, along with their chemical reaction products, are transported and deposited away from their sources. $NO_x$ is a short-lived gas with a lifetime of 1 to 10 days. Airborne $NO_x$ is involved in acidification and ozone production, but is also of great concern because of its toxicity to humans, animals, and plants. $NH_3$ has a lifetime of only a few hours to a few days. It is the primary acid neutralizing agent in the atmosphere, affecting the pH of aerosols, cloud water, and rainfall, but can be also an acidifying agent in nitrification processes[4].

### Nitrogen Dioxide

While the first emission inventory on $SO_2$, based on 1989 data, was completed in 1992 for the Rijeka Bay area[7], the first emission inventory on $NO_2$ was completed only recently[8], although emissions of $NO_2$ from four major industrial plants (based on 1989 data) were estimated earlier[9].

In the only complete emission inventory, in 1995 the total emission of $NO_2$ was estimated to 3627 t/a (1104 t N/a). The contribution of three major industrial sources (the old and new petroleum refineries and the power plant) was 2727 t/a (830 t N/a), i.e., 75.2% of the total, while other industrial sources contributed 76 t/a (23 t N/a), i.e., 2.1%. The new petroleum refinery and the power plant remained the biggest sources of $NO_2$ emissions in the city and the eastern industrial area (traffic is

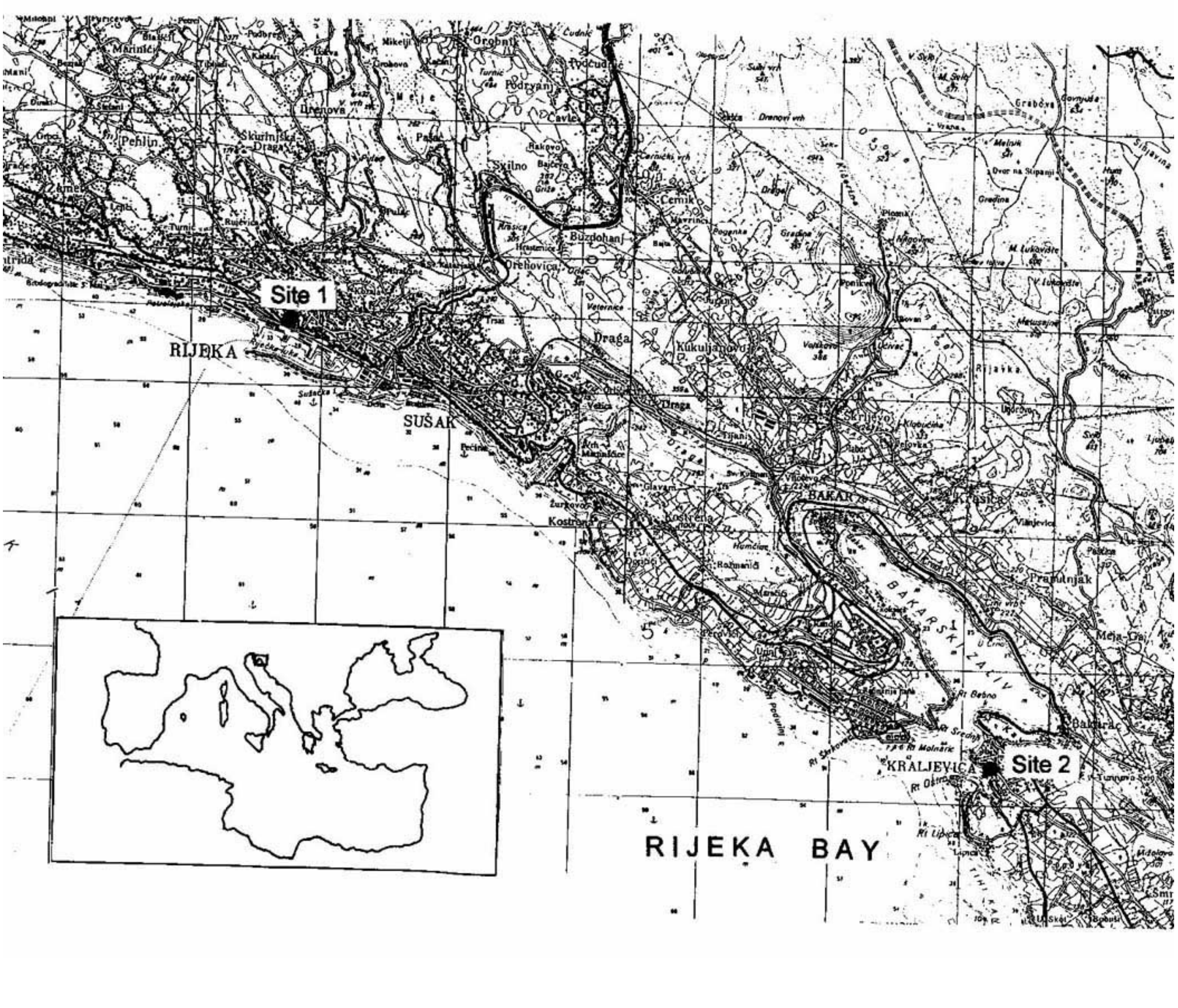

**FIGURE 1.** Location of sampling sites.

dominant in the rest of the region, and in the whole county area generally). Emission of $NO_2$ from traffic was estimated at 705 t/a (215 t N/a), forming 19.4% of the total, while the contribution of other sources was equal to 119 t/a (36 t N/a), i.e., 3.3%. According to 1989 data (4686 t/a = 1426 t N/a), emission of $NO_2$ from major industrial sources was reduced by 40%. Such a marked reduction in emission should result in a decrease in ambient levels of $NO_2$. Annual means of $NO_2$ and $NH_3$, together with corresponding trendlines, are given in Fig. 2 (for Site 1) and Fig. 3 (for Site 2). For comparative reasons, ambient levels of $SO_2$ are also presented.

While ambient levels of $SO_2$ show substantial decrease since 1989 at both sites (66% at Site 1 and 55% at Site 2), reflecting the 70% reduction in emissions from four major industrial sources[10], trends of $NO_2$ and $NH_3$ do not follow the reduced emissions of these pollutants. Annual means of $NO_2$ were in the range of 34 to 60 μg/m$^3$ at Site 1 (often exceeding the national limit value of 40 μg/m$^3$) and 14 to 26 μg/m$^3$ at Site 2. Maximum daily concentrations were mostly above 100 μg/m$^3$ (maximum 169 μg/m$^3$) at Site 1, and between 40 and 90 μg/m$^3$ at Site 2. Annual means of $NO_2$ at urban Site 1 were in the range of the corresponding values observed in 1987 in some U.K. cities (except London), while maximum daily concentrations were several times lower than the maxima observed in the U.K.[11]. The obtained yearly averages at both sites are in the range of the ambient levels of $NO_2$ observed in Denmark. While no difference in $NO_2$ for the summer and winter results were obtained in Denmark[12], the winter results were found to be higher in Great Britain[11]. During the last 5 years of survey (Table 1), seasonal variations are visible only once at Site 1, presumably due to dominant traffic emissions from the nearby busy street, and in 3 of 5 years at Site 2.

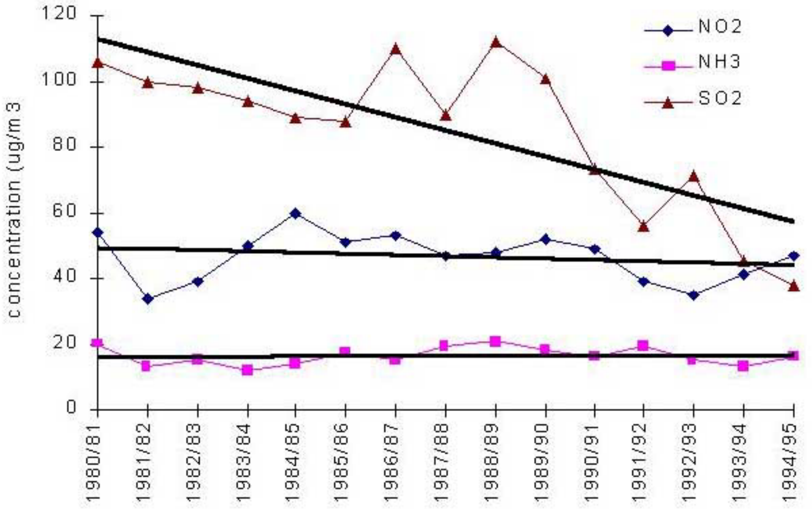

**FIGURE 2.** Annual means of $SO_2$, $NO_2$, and $NH_3$ at urban Site 1.

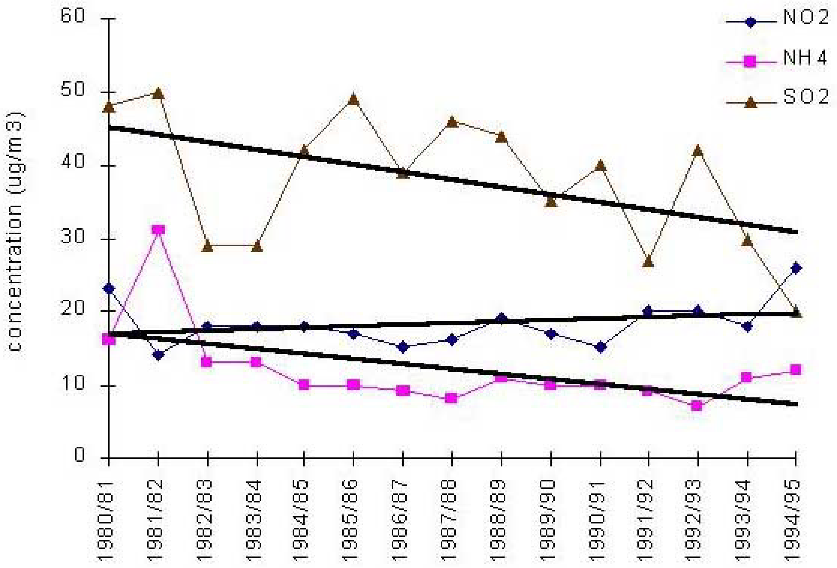

**FIGURE 3.** Annual means of $SO_2$, $NO_2$, and $NH_3$ at suburban Site 2.

**TABLE 1**
**Seasonal $NO_2$ Concentrations (1990/91–1994/95)**

| | Site 1 | | | Site 2 | | |
|---|---|---|---|---|---|---|
| Year | Winter | Summer | W/S | Winter | Summer | W/S |
| 1990/91 | 50 | 47 | 1,06 | 18 | 13 | 1,42 |
| 1991/92 | 39 | 41 | 0,94 | 21 | 20 | 1,09 |
| 1992/93 | 35 | 36 | 0,99 | 21 | 20 | 1,03 |
| 1993/94 | 39 | 43 | 0,90 | 20 | 14 | 1,41 |
| 1994/95 | 47 | 38 | 1,21 | 27 | 20 | 1,33 |

In spite of several ups and downs, the annual means of $NO_2$ at Site 1 show a decrease during the period studied. In contrast, a slight increase is observed for the same period at Site 2. Annual means of $NO_2$ exceeded those of $SO_2$ at both sites in 1995.

## Ammonia

In 1989, industrial emission of $NH_3$ was estimated only for the coke plant, though other industrial plants located in the Rijeka Bay area are also potential sources of industrial $NH_3$. The estimated value of 993 to 1329 t/a (818 to 1094 t N/a) is mainly due to fugitive emissions from the coke battery due to poor maintenance[9]. Though data on $NH_3$ emissions from traffic and other industrial sources are lacking, after the closure of the coke plant a decline in ambient levels of $NH_3$ would be expected (but has not yet seen in the period analysed). Due to the shortage of fertile soil in the region, agricultural activities are hardly to be considered as a significant source of $NH_3$ emissions. Because of various emission factors for humans[2], emission of $NH_3$ from the population living in the wider city area is estimated to be between 51 and 316 t/a (42 to 260 t N/a). Taking into account these values, an average $NH_3$ emission density between 3700 and 5850 kg/km$^2$ is obtained for the Rijeka Bay area. These values are close to and/or even higher than the corresponding value obtained in the Netherlands (4000 kg/km$^2$), known to be the highest in Europe[13]. The lower estimate could be more realistic since ambient levels of $NH_3$ are at the same level as those determined in those areas of the Netherlands affected by $NH_3$ emissions[13]. Subsequent to the coke plant's shutdown, the average emission density should be considerably reduced.

Annual means of $NH_3$ are in the range of 13 to 21 µg/m$^3$ at Site 1 and 7 to 16 µg/m$^3$ at Site 2, if the yearly average for 1981/82 (31 µg/m$^3$) is excluded. Annual means are several times lower than the values obtained in the vicinity of the petrochemical plant with fertilizer production (40 to 80 µg/m$^3$) in continental Croatia[14], but also several times higher than the results obtained in the Philadelphia urban area[3]. Except for the initial decline in ambient levels of $NH_3$ at Site 2, giving rise to the overall decline trend, the ambient levels of $NH_3$ are almost constant at both sites. Higher yearly averages of $NO_2$ and $NH_3$ at urban Site 1 are due to the smaller distance between sources of these pollutants (refinery, traffic), higher population density, complex and unfavourable orography, and less exposure to dominant northeasterly winds compared to Site 2.

## CONCLUSION

Industrial plants within the Rijeka Bay area represent the major air pollution sources regarding $SO_2$ (95% of total), $NO_2$ (77% of total), and $NH_3$. Reduction by 70% of $SO_2$ emissions since 1989 resulted in a decline in the ambient levels of this pollutant observed at both sites. In spite of reduction in industrial $NO_2$ emissions by 40%, the ambient levels of $NO_2$ do not follow the cited reduction. A possible reason for such behaviour is the dominant impact of minor $NO_2$ sources like traffic at these sampling sites. The same stands for ambient levels of $NH_3$, in spite of the fact that the coke plant ceased its production at the end of 1994. A possible involvement of nitrogen compounds in complex atmospheric chemistry could also be considered as a possible cause of a lack of downward trends in ambient levels of nitrogen gaseous pollutants.

| | | |
|---|---|---|
| Received: | July | 19, 2001 |
| Revised: | September | 14, 2001 |
| Accepted: | October | 5, 2001 |
| Published: | October | 26, 2001 |

## BIOSKETCH

**Ana Alebić–Juretić** is Head of the Air Pollution Division at The Institute of Public Health, Rijeka, Croatia. She holds a Ph.D. in Chemistry. Her research is focused on atmospheric chemistry, environmental sciences, and toxicology. Dr. Alebic-Juretic's main research interest is air pollution within Kvarner Bay area (Croatia), and consequently harmful effects of such pollution on plants (lichens) and human (reproductive) health in the same area. Another subject of research is the heterogeneous ozone reactions under simulated atmospheric conditions and photoacoustic pectroscopy of powdered samples. Her research results have been published in approximately 50 papers and submitted to highly qualified journals.
